# Use of the Patient-Reported Outcomes Measurement Information System (PROMIS) Outcome Measures in Lumbar Decompression Surgery: A Systematic Review

**DOI:** 10.7759/cureus.105726

**Published:** 2026-03-23

**Authors:** Harneet K Cheema, Manraj S Cheema, James Gomes

**Affiliations:** 1 Health Sciences, University of Ottawa, Ottawa, CAN

**Keywords:** degenerative lumbar disease, lumbar decompression, lumbar spine surgery, microdiscectomy, patient-reported outcomes, patient-reported outcomes measurement information system, physical function, promis, systematic review

## Abstract

Degenerative lumbar spine diseases are a major cause of disability and may require surgical intervention when conservative management fails. Patient-reported outcome measures are commonly used to assess postoperative recovery following lumbar decompression. Traditional instruments such as the Oswestry Disability Index (ODI), Visual Analog Scale (VAS), and Short Form Health Surveys are widely used but have limitations, including fixed length and ceiling effects. The Patient-Reported Outcomes Measurement Information System (PROMIS) was developed to address these limitations through standardized domains and adaptive testing. Although PROMIS has increasingly been used in spine surgery research, the literature on lumbar decompression remains heterogeneous. A systematic review was conducted according to the PRISMA 2020 guidelines to evaluate the use of PROMIS outcome measures in lumbar decompression surgery. PubMed, Embase, and Scopus were searched for English-language studies published between January 2010 and January 2026. Eligible studies included adult patients undergoing lumbar decompression for degenerative lumbar spine conditions reporting PROMIS outcomes. Extracted data included study characteristics, PROMIS domains, follow-up duration, analytical approaches, and key findings related to postoperative responsiveness, clinically meaningful improvement, predictive utility, and correlations with legacy outcome measures. Due to heterogeneity in PROMIS domains, follow-up intervals, and analytical methods, results were synthesized qualitatively. Forty-five studies comprising 13,444 patients met the inclusion criteria. Most studies were retrospective cohort studies (43/45), conducted in the United States (43/45), and performed at single institutions (41/45). PROMIS Physical Function (PF) was the most frequently evaluated domain, appearing in 42 studies. Pain-related domains such as pain interference and pain intensity were also commonly assessed, while psychological and global health domains were evaluated less frequently. Overall, 40 studies (88.9%) reported postoperative improvement in at least one PROMIS domain following lumbar decompression. Minimal clinically important difference (MCID) analyses were reported in 32 studies (71.1%). Sixteen studies (35.6%) evaluated PROMIS measures as predictors of postoperative outcomes, while 11 studies (24.4%) reported correlations with legacy outcome instruments such as ODI and VAS. PROMIS PF consistently demonstrated responsiveness to postoperative improvement and moderate to strong correlations with traditional outcome measures. PROMIS outcome measures appear to be a useful framework for evaluating recovery following lumbar decompression surgery. PROMIS PF and pain-related domains demonstrate responsiveness to postoperative improvement and show moderate to strong correlations with established legacy instruments. Emerging evidence suggests that PROMIS scores may have potential prognostic value in assessing postoperative recovery trajectories. However, the current literature is characterized by substantial heterogeneity in PROMIS domain selection, MCID reporting, and analytical methodology, and is predominantly based on retrospective cohort studies. As such, these findings should be interpreted in the context of these limitations, and further methodological standardization and prospective research are needed to better define the role of PROMIS in lumbar spine surgery outcome assessment.

## Introduction and background

Degenerative lumbar spine diseases represent a significant cause of disability worldwide [1] and may require surgical intervention when conservative treatment methods fail [2]. This burden exists within the broader context of low back pain, which ranks highest among all conditions for years lived with disability globally [1]. Degenerative conditions such as lumbar disc degeneration, lumbar spinal stenosis, and degenerative spondylolisthesis commonly cause back pain, radiculopathy, and daily functional limitations that substantially impair quality of life [3]. When conservative management strategies do not succeed, lumbar decompression procedures, including open and minimally invasive techniques, are performed to relieve neural compression and improve patient outcomes [3,4].

The accurate evaluation of surgical outcomes in spine surgery has increasingly relied on patient-reported outcome measures (PROMs), which provide information regarding symptom burden, functional limitation, and health-related quality of life from a patient’s perspective [5]. Traditionally, legacy outcome instruments have been used to assess patient recovery following lumbar spine surgery [5]. These legacy outcome instruments include the Oswestry Disability Index (ODI), the Visual Analog Scale (VAS) for pain, and Short Form (SF) health surveys such as the SF-12 and SF-36 [5,6]. Although these traditional methods remain useful, they also possess several limitations, including fixed questionnaire length, ceiling and floor effects, and limited comparability across various studies and clinical populations [7].

The Patient-Reported Outcomes Measurement Information System (PROMIS) was developed by the National Institutes of Health with the purpose of addressing many of these limitations through the use of standardized outcome domains and computer adaptive testing methods [8]. These PROMIS instruments evaluate a range of health dimensions, including physical function, pain interference, and mental health domains such as depression and anxiety [9]. Through computerized adaptive testing, which tailors questions based on prior responses, these instruments reduce respondent burden while maintaining measurement precision [9]. The PROMIS system has increasingly been used in spine surgery research, where PROMIS Physical Function (PF) has demonstrated sound psychometric properties in patients with spinal disorders [7]. In a large spine cohort, PROMIS PF computer adaptive testing demonstrated improved measurement coverage, reduced floor and ceiling effects, and lower administration burden compared with the ODI and the SF-36 physical function domain [10]. In addition, PROMIS PF and Pain Interference (PI) have shown strong responsiveness to clinical change across multiple follow-up time points in spine patients [11,12], and PROMIS measures have demonstrated moderate to strong correlations with legacy instruments such as the ODI and Neck Disability Index (NDI), supporting convergent validity and clinical interpretability in spine populations [13].

However, despite these established strengths, the application of PROMIS in lumbar decompression surgery remains heterogeneous across studies. Beyond assessing postoperative change, PROMIS scores have been evaluated as predictors of surgical outcomes, with psychosocial factors and baseline symptom severity influencing postoperative trajectories in several cohorts [12]. These capabilities may be particularly relevant in lumbar decompression surgery, where postoperative recovery is heterogeneous and involves multidimensional changes in pain, physical function, and psychosocial status that may not be fully captured by legacy region-specific instruments alone.

Despite increasing use of PROMIS in spine surgery, the current literature evaluating PROMIS outcomes following lumbar decompression remains limited and inconsistently reported. Existing studies vary in the PROMIS domains assessed, the definitions and thresholds used to determine clinically meaningful improvement, and the analytical approaches used to evaluate postoperative responsiveness and predictive utility. Although prior reviews have summarized the use of PROMIS in broader spine populations, a focused synthesis of PROMIS performance specifically in lumbar decompression surgery is lacking. This lack of procedural specificity and methodological standardization limits the ability to interpret findings across studies and to define the role of PROMIS relative to legacy outcome measures in this population. Accordingly, this study aimed to provide a comprehensive synthesis of how PROMIS outcome measures have been used in patients undergoing lumbar decompression surgery, with a focus on postoperative responsiveness, clinically meaningful improvement, predictive utility, and correlations with legacy outcome measures.

## Review

Methods

Study Design and Reporting

This systematic review was conducted to synthesize the current evidence on the use of the PROMIS in patients undergoing lumbar decompression surgery. The objective was to evaluate how PROMIS instruments have been applied to assess surgical outcomes in this population and to summarize the evidence regarding their postoperative responsiveness, clinically meaningful change, predictive utility, and correlation with legacy outcome measures across the existing literature. The review was conducted and reported in accordance with the Preferred Reporting Items for Systematic Reviews and Meta-Analyses (PRISMA) 2020 guidelines. A formal review protocol was not registered prior to conducting the study. This was due to the exploratory nature of the review and the iterative development of the research question during the early stages of study identification and eligibility refinement. However, we acknowledge that protocol registration is recommended to enhance transparency and minimize potential reporting bias, and the absence of a pre-registered protocol represents a methodological limitation of this study.

Information Sources and Search Strategy

A comprehensive search was performed in PubMed, Embase, and Scopus to identify relevant studies. The search was restricted to English-language studies involving adult human populations published between January 2010 and January 2026. The final database search was conducted on February 2nd, 2026.

Search strategies were developed using a keyword-based approach to capture studies evaluating the use of the PROMIS in lumbar decompression surgery. Core concepts included PROMIS, lumbar spine pathology, and decompression procedures. To improve sensitivity and ensure comprehensive retrieval of relevant studies, multiple synonymous terms were incorporated for lumbar pathology (e.g., “lumbar spine,” “lumbar disc,” and “spinal stenosis”) and surgical procedures (e.g., “decompression,” “laminectomy,” “discectomy,” and “microdiscectomy”) (Table 1).

**Table 1:** Summary of database-specific search strategy. Table 1 provides a simplified overview of the core search concepts, while full database-specific search strategies are provided in Appendices A-C to ensure reproducibility.

Database	Search details
PubMed	(PROMIS OR "Patient-Reported Outcomes Measurement Information System") AND (lumbar OR "lumbar spine" OR "lumbar disc" OR "spinal stenosis") AND (decompression OR laminectomy OR discectomy OR microdiscectomy) Filters applied: Humans, Adults (≥18 years), English language, 2010-2026
Embase	(PROMIS OR "Patient-Reported Outcomes Measurement Information System") AND (lumbar OR "lumbar spine" OR "lumbar disc" OR "spinal stenosis") AND (decompression OR laminectomy OR discectomy OR microdiscectomy) Filters applied: Humans, English language, 2010-2026
Scopus	TITLE-ABS-KEY (PROMIS OR "Patient-Reported Outcomes Measurement Information System") AND TITLE-ABS-KEY (lumbar OR "lumbar spine" OR "lumbar disc" OR "spinal stenosis") AND TITLE-ABS-KEY (decompression OR laminectomy OR discectomy OR microdiscectomy) Filters applied: Humans, English language, 2010- 2026

Search terms were applied using database-specific syntax as supported by each platform. In PubMed, searches were conducted using keyword-based queries mapped to all fields and combined with database filters. In Embase, searches were performed using the database’s multi-purpose (.mp.) field. In Scopus, searches were restricted to title, abstract, and keyword fields using the TITLE-ABS-KEY function. Controlled vocabulary terms (e.g., MeSH in PubMed or Emtree in Embase) were not used as core conceptual search terms. However, in PubMed, the platform’s adult filter was applied in addition to the keyword-based search strategy. Instead, a keyword-based approach was used to allow consistent application of search terms across databases and to capture variations in terminology used in the literature. While the inclusion of controlled vocabulary may further enhance search sensitivity, a keyword-based strategy was selected to maintain consistency and transparency across database searches.

Database-specific filters were applied where available. Across all databases, searches were limited to human studies, English-language publications, and the specified publication date range. In PubMed, an adult filter (≥18 years) was additionally applied. In Embase and Scopus, where an equivalent adult filter was not uniformly available, adult eligibility was enforced during the screening process according to the predefined inclusion criteria.

The full electronic search strategies for PubMed, Embase, and Scopus are provided in Appendices A-C. Reference lists of all included studies were manually screened to identify additional relevant articles.

Eligibility Criteria

Studies were eligible if they enrolled adult human participants (≥18 years) undergoing lumbar decompression surgery for degenerative lumbar spine conditions and reported postoperative PROMIS outcomes. Eligible studies examined conditions such as lumbar disc herniation, lumbar spinal stenosis, degenerative spondylolisthesis, and mixed degenerative lumbar pathology. All studies were required to report at least one PROMIS outcome measure. Eligible studies included domains such as physical function, pain interference, pain intensity, pain behavior, depression, anxiety, sleep disturbance, and global health.

Studies were excluded from the review if they exclusively examined lumbar fusion procedures. Studies including mixed decompression and fusion cohorts were included only if decompression-specific outcomes were reported separately; otherwise, they were excluded. Additionally, any studies that only evaluated nonoperative management, such as physical therapy or injections, were excluded. Studies that examined patients with nondegenerative spinal conditions, including trauma, infection, tumor, deformity, or fracture, were excluded as well. Further, studies that focused on cervical or thoracic spine procedures were excluded, unless lumbar decompression outcomes were separately reported.

Only original research studies were eligible for inclusion in this review, including retrospective cohort studies, prospective cohort studies, and randomized controlled trials. Narrative reviews, systematic reviews, editorials, letters, case reports, conference abstracts without corresponding full texts, and study protocols were excluded.

Study Selection

**Figure 1:**
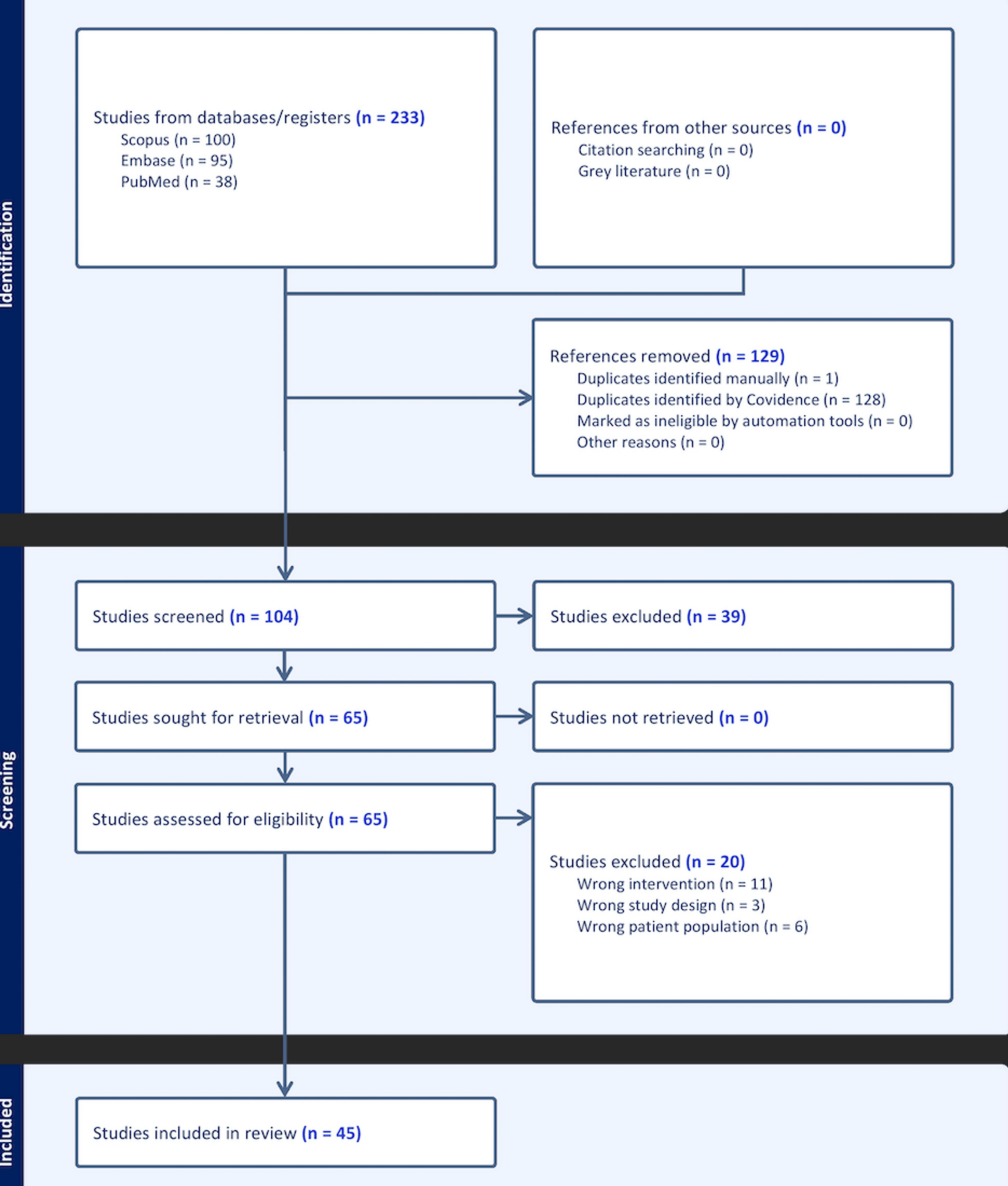
PRISMA flow diagram of the study selection process. PRISMA: Preferred Reporting Items for Systematic Reviews and Meta-Analyses.

Prior to screening, all records identified through database searches were exported onto Covidence (Melbourne, Australia), and duplicates were removed. Study selection was performed in two stages. Two reviewers independently screened titles and abstracts for potential eligibility, and any studies that did not meet the inclusion criteria were excluded at this stage. The full texts of all remaining records were then retrieved and independently reviewed by both reviewers to determine the final eligibility, based on predefined inclusion and exclusion criteria. Any disagreements were resolved through discussion and consensus. The selection process was documented using a PRISMA flow diagram (Figure 1).

Data Extraction

Data extraction was conducted independently by two reviewers using a pre-determined standardized data extraction form that was piloted on a subset of included studies to ensure consistency and completeness. Extracted data included study characteristics (authors, year of publication, country, study setting, study design, and single versus multicenter status), sample size, and patient population. Surgical characteristics were also recorded, including the type of lumbar decompression procedure performed and the underlying degenerative spinal pathology. Information regarding PROMIS utilization was also extracted for each included study, including the PROMIS domains evaluated and whether these PROMIS measures were used as primary outcome measures, predictors, or for validity testing against legacy outcome instruments. Information regarding follow-up periods was also collected, divided into short-term (less than six months), mid-term (between six and 12 months), and long-term (more than 12 months) follow-up categories. Moreover, the analytical approaches used to evaluate PROMIS outcomes were also extracted for this review. These included evaluations of postoperative improvement in PROMIS domains, analyses of minimal clinically important difference (MCID), correlations between PROMIS scores and legacy outcome measures, evaluation of PROMIS measures as predictors of postoperative outcomes, and the identification of factors associated with variations in PROMIS improvement across patient populations. Additionally, the key findings pertaining to PROMIS performance were also extracted for each study.

Following the extraction, two reviewers compared their extracted data for completeness and accuracy. The reviewers resolved all discrepancies through discussion and consensus.

Risk of Bias Assessment

Risk of bias was assessed independently by two reviewers using the Newcastle-Ottawa Scale (NOS) for cohort studies [14]. The NOS evaluates methodological quality across three domains: selection of study groups (0-4 points), comparability of cohorts (0-2 points), and outcome assessment (0-3 points), with a maximum score of nine points [14]. Studies were categorized as low quality (0-3), moderate quality (4-6), or high quality (7-9) based on their total NOS score [14]. Any discrepancies in scoring were resolved through discussion and consensus.

Data Synthesis and Statistical Analysis

As a result of substantial heterogeneity across the included studies in terms of PROMIS domains assessed, follow-up durations, outcome reporting methods, and analytical approaches, quantitative pooling of results was not performed. Heterogeneity was assessed qualitatively by comparing key study characteristics, including differences in PROMIS domains evaluated, timing of outcome assessment (short-, mid-, and long-term follow-up), definitions and thresholds of clinically meaningful improvement (e.g., MCID), and the analytical approaches used to evaluate PROMIS outcomes across studies.

Given the variability in these domains, as well as differences in study design and patient populations, the included studies were not considered sufficiently comparable to support meta-analysis. Accordingly, a qualitative synthesis was conducted.

For the purpose of synthesis, findings were organized and reported according to key thematic domains, including: (1) PROMIS domain utilization, (2) postoperative responsiveness of PROMIS measures, (3) clinically meaningful improvement (MCID) and clinical interpretability, (4) correlations with legacy outcome measures, and (5) predictive utility and factors associated with variability in outcomes. Within each domain, results were summarized descriptively, with emphasis on patterns in PROMIS utilization, reported postoperative changes, and the range of analytical approaches used across studies.

Given this heterogeneity and the absence of comparable effect estimates across studies, statistical measures of heterogeneity (e.g., I² statistics) were not calculated.

Results

Study Characteristics

A total of 45 studies met the inclusion criteria [15-59], comprising 13,444 patients undergoing lumbar decompression procedures. The included studies were predominantly retrospective cohort designs conducted in the United States and primarily at single institutions, with only a small number representing multicenter or registry-based analyses. Detailed study characteristics, including study design, patient populations, surgical procedures, and follow-up durations, are summarized in Table 2.

**Table 2:** Characteristics of included studies evaluating PROMIS outcomes following lumbar decompression surgery. PROMIS: Patient-Reported Outcomes Measurement Information System; PF: Physical Function; PI: Pain Interference; PB: Pain Behavior. Follow-up definitions: short-term <6 months; mid-term = 6-12 months; long-term >12 months.

Author (year)	Country	Study design	Sample size (n)	Procedure type	Degenerative indication	PROMIS domains used	Follow-up duration
Anwar et al. (2024) [15]	USA	Retrospective cohort	347	Minimally invasive decompression	Mixed degenerative pathology	PF, PI, Anxiety, Sleep Disturbance	Short + long-term
Anwar et al. (2024) [16]	USA	Retrospective cohort	421	Lumbar decompression	Mixed degenerative pathology	PF	Short + long-term
Anwar et al. (2024) [17]	USA	Retrospective cohort	115	Minimally invasive decompression	Mixed degenerative pathology	PF, Anxiety, Sleep Disturbance	Short + mid + long-term
Anwar et al. (2025) [18]	USA	Retrospective cohort	102	Lumbar decompression	Disc herniation	PI	Short + mid + long-term
Bhatt et al. (2019) [19]	USA	Prospective cohort	78	Microdiscectomy	Disc herniation	PF, PI, PB	Short-term
Bovonratwet (2022) [20]	USA	Retrospective cohort	79	Minimally invasive decompression	Degenerative spondylolisthesis	PF	Short + mid + long-term
Bovonratwet (2024) [21]	USA	Retrospective cohort	127	Microdiscectomy	Disc herniation	PF	Short + mid + long-term
Cha et al. (2021) [23]	USA	Retrospective cohort	216	Minimally invasive decompression	Mixed degenerative pathology	PF	Short + mid-term
Cha et al. (2021) [24]	USA	Retrospective cohort	92	Minimally invasive decompression	Mixed degenerative pathology	PF	Short + mid + long-term
Cha et al. (2021) [58]	USA	Retrospective cohort	91	Minimally invasive decompression	Mixed degenerative pathology	PF	Short + mid-term
Cha et al. (2022) [22]	USA	Retrospective cohort	811	Minimally invasive decompression	Mixed degenerative pathology	PF	Short + mid-term
Chen et al. (2021) [25]	China	Retrospective cohort	25	Endoscopic decompression	Mixed degenerative pathology	PF, PI	Short + mid-term
Chen et al. (2022) [26]	China	Retrospective cohort	21	Endoscopic decompression	Disc herniation	PF, PI	Short + mid-term
Jacob et al. (2023) [28]	USA	Retrospective cohort	216	Minimally invasive decompression	Mixed degenerative pathology	PF	Short + mid + long-term
Jacob et al. (2024) [27]	USA	Retrospective cohort	129	Minimally invasive decompression	Mixed degenerative pathology	PF	Short + mid + long-term
Karhade et al. (2021) [29]	USA	Retrospective cohort	906	Lumbar decompression	Mixed degenerative pathology	PF, PI, Pain Intensity	Short + mid-term
Karhade et al. (2021) [30]	USA	Prospective cohort	636	Lumbar decompression	Mixed degenerative pathology	PF	Mid-term
Kasir et al. (2025) [31]	USA	Retrospective cohort	2408	Microdiscectomy	Disc herniation	PF	Short + mid + long-term
Khechen et al. (2018) [32]	USA	Retrospective cohort	41	Minimally invasive microdiscectomy	Disc herniation	PF	Short + mid-term
Korsun et al. (2024) [33]	USA	Retrospective cohort	390	Minimally invasive decompression	Lumbar stenosis	PF	Short + mid + long-term
Lightsey et al. (2022) [34]	USA	Retrospective cohort	35	Minimally invasive decompression	Disc herniation	PF, PI, Pain Intensity, Depression, Anxiety, Global Health	Short + mid + long-term
Lynch et al. (2021) [35]	USA	Retrospective cohort	402	Minimally invasive microdiscectomy	Mixed degenerative pathology	PF	Short + mid + long-term
Lynch et al. (2022) [36]	USA	Retrospective cohort	453	Minimally invasive decompression	Mixed degenerative pathology	PF	Short + mid + long-term
Mazur-Hart et al. (2025) [37]	USA	Retrospective cohort	176	Lumbar decompression	Lumbar stenosis	PF	Mid-term
Merrill et al. (2018) [38]	USA	Retrospective cohort	111	Mixed decompression procedures	Lumbar stenosis	PF, Pain Intensity, Depression	Mid-term
Mohanty et al. (2023) [39]	USA	Retrospective cohort	340	Mixed decompression procedures	Mixed degenerative pathology	Global Health	Short + mid-term
Nie et al. (2023) [40]	USA	Retrospective cohort	343	Minimally invasive decompression	Mixed degenerative pathology	PF	Short + mid + long-term
Nie et al. (2023) [41]	USA	Retrospective cohort	107	Lumbar decompression	Mixed degenerative pathology	PF, PI, Anxiety, Sleep Disturbance	Short + mid-term
Nie et al. (2023) [42]	USA	Retrospective cohort	87	Lumbar decompression	Mixed degenerative pathology	PF, PI, Anxiety, Sleep Disturbance	Short + mid-term
Nie et al. (2023) [43]	USA	Retrospective cohort	473	Lumbar decompression	Mixed degenerative pathology	PF, PI, Anxiety, Sleep Disturbance	Short + long-term
Nolte et al. (2021) [44]	USA	Retrospective cohort	135	Minimally invasive decompression	Mixed degenerative pathology	PF	Short + mid-term
Nolte et al. (2021) [59]	USA	Retrospective cohort	314	Lumbar decompression	Lumbar stenosis	PF	Short + mid-term
Oris et al. (2026) [45]	USA	Retrospective cohort	152	Lumbar decompression	Mixed degenerative pathology	PF, PI	Mid-term
Parrish et al. (2022) [46]	USA	Retrospective cohort	351	Lumbar decompression	Mixed degenerative pathology	PF	Short + mid-term
Roca et al. (2024) [47]	USA	Retrospective cohort	182	Lumbar decompression	Mixed degenerative pathology	PF, PI, Anxiety, Sleep Disturbance	Short + mid-term
Rubery et al. (2019) [48]	USA	Retrospective cohort	78	Microdiscectomy	Disc herniation	PF, PI, Depression	Short-term
Shahi et al. (2022) [49]	USA	Retrospective cohort	345	Minimally invasive decompression	Lumbar stenosis	PF	Short + mid + long-term
Song et al. (2024) [50]	USA	Retrospective cohort	163	Microdiscectomy	Disc herniation	PF	Short + mid + long-term
Turcotte et al. (2023) [51]	USA	Retrospective cohort	342	Mixed decompression procedures	Lumbar stenosis	PF	Short-term
Varlotta et al. (2020) [52]	USA	Retrospective cohort	88	Microdiscectomy	Disc herniation	PF, PI, Pain Intensity	Short-term
Virk et al. (2020) [53]	USA	Retrospective cohort	126	Microdiscectomy	Disc herniation	Global Health	Short + mid-term
Virk et al. (2020) [54]	USA	Retrospective cohort	138	Minimally invasive microdiscectomy	Disc herniation	PF	Short + mid-term
Ward et al. (2025) [55]	USA	Retrospective cohort	82	Lumbar decompression	Mixed degenerative pathology	PF	Short + mid + long-term
Wolf et al. (2024) [56]	USA	Retrospective cohort	277	Lumbar decompression	Lumbar stenosis	PF	Short + mid + long-term
Zhang et al. (2023) [57]	USA	Retrospective cohort	893	Lumbar decompression	Mixed degenerative pathology	PF, PI, Depression, Anxiety	Short + mid + long-term

Surgical procedures examined lumbar decompression without fusion, minimally invasive decompression techniques, microdiscectomy, endoscopic decompression, and mixed decompression procedures. Lumbar decompression (open or unspecified approach) and minimally invasive decompression were the most commonly studied procedures, followed by microdiscectomy. Endoscopic decompression and mixed decompression procedures were among the least frequently represented.

The degenerative indications were heterogeneous. Most of the included studies examined mixed degenerative lumbar pathology, commonly including combinations of lumbar disc herniation and lumbar spinal stenosis. Disc herniation-specific and stenosis-specific cohorts were well represented in the literature, while degenerative spondylolisthesis was less commonly investigated as the primary indication.

Follow-up durations varied across studies, with most cohorts reporting early postoperative outcomes, commonly at six- to 12-week intervals, and a subset incorporating mid-term (6-12 months) and long-term (>12 months) follow-up. Early postoperative assessments were the most consistently reported across studies, whereas longer-term follow-up was less uniformly available. Overall patterns of follow-up timing are summarized in Table 2.

PROMIS Domain Utilization

PROMIS Physical Function (PF) was the predominant domain assessed in the studies, appearing in 42 of 45 studies either alone or in combination with other PROMIS domains. Pain-related domains were the second most frequently evaluated, including pain interference, pain intensity, and pain behavior. Mental health-related domains, including depression and anxiety, were reported less consistently across the literature. Sleep disturbance and global health measures were also evaluated in a minority of studies.

The majority of investigations focused on a limited subset of PROMIS domains, commonly PF alone or PF combined with PI. Only a smaller proportion of studies included both physical and psychological constructs. Particularly, mental health domains were typically examined in combination with physical function rather than as primary outcomes. This pattern demonstrates an emphasis on functional recovery and pain reduction in the lumbar decompression literature, with comparatively less comprehensive evaluation of broader biopsychosocial health domains. Additionally, PROMIS was administered to patients using a mix of computer adaptive testing (CAT) and short-form instruments across studies.

Postoperative Responsiveness of PROMIS

Across the included studies, PROMIS domains demonstrated consistent postoperative improvement following lumbar decompression. Of the 45 studies, 40 (88.9%) reported postoperative improvement in at least one PROMIS domain after surgery. PROMIS PF was the most consistently responsive domain, with the majority of studies demonstrating significant improvement from baseline across early, mid-term, and long-term follow-up intervals. Pain-related domains, including PROMIS Pain Interference (PI), Pain Intensity, and Pain Behavior (PB), also showed robust postoperative improvement in most cohorts in which they were assessed.

Although reporting formats varied across the studies, several investigations provided quantitative estimates of improvement. Reported mean PROMIS-PF improvements ranged approximately from 8 to 13 points within early follow-up periods, with one study demonstrating an increase from 37.4 preoperatively to 50.3 at 12 weeks. Similarly, PROMIS PI decreased by approximately 10-11 points in some early postoperative assessments. Together, these findings support substantial postoperative responsiveness of PROMIS domains following lumbar decompression.

Clinically meaningful improvement, as measured by MCID, was reported in 32 of 45 studies (71.1%). Across these studies, MCID achievement rates for PROMIS PF generally ranged from approximately 60% to over 80% at mid- to long-term follow-up, with several studies reporting rates exceeding 75% within one year. Pain-related domains demonstrated similar patterns of clinically meaningful improvement, further supporting the sensitivity of PROMIS instruments in capturing postoperative recovery.

Mental health domains such as PROMIS Depression, Anxiety, and Sleep Disturbance were evaluated less frequently; however, when assessed, postoperative improvement was commonly observed. Improvements in psychological domains were often more pronounced in cohorts with higher baseline symptom burden, suggesting that patients with greater preoperative impairment experienced larger absolute gains. Postoperative improvement was often evident as early as six weeks and, in many cohorts, was sustained through six to 12 months and up to two years. Several investigations noted convergence of outcomes across baseline severity strata, wherein patients with worse preoperative PROMIS scores demonstrated a greater magnitude of improvement yet achieved comparable postoperative scores relative to less impaired cohorts. In several studies, postoperative improvement was evaluated through MCID attainment rather than formal statistical testing, reflecting an emphasis on clinically meaningful recovery. A minority of studies (five of 45) did not formally assess the statistical significance of baseline-to-postoperative change or focused primarily on predictive modeling rather than longitudinal change analysis. The overall methodological utilization of PROMIS outcomes across the included studies is summarized in Table 3.

**Table 3:** Summary of PROMIS outcome analyses across included studies. PROMIS: Patient-Reported Outcomes Measurement Information System; MCID: minimal clinically important difference.

Outcome category	Number of studies (n = 45)	Percentage (%)
Reported significant postoperative PROMIS improvement	40	88.9
Evaluated MCID	32	71.1
Assessed correlation with legacy measures	11	24.4
Performed predictive modeling	16	35.6
Identified modifiers of improvement	23	51.1

Risk of Bias Assessment

Risk-of-bias assessment using the NOS is summarized in Table 4. Overall, the included studies demonstrated moderate methodological quality. A substantial proportion of studies were classified as moderate risk of bias, with several studies achieving high-quality scores. Lower scores were most commonly attributable to limited comparability between cohorts and the predominance of retrospective study designs. Selection and outcome assessment domains were generally well addressed across studies.

**Table 4:** Newcastle-Ottawa Scale quality assessment of included studies.

Study	Selection (0-4)	Comparability (0-2)	Outcome (0-3)	Total	Quality
Anwar et al. (2024) [15]	3	1	1	5/9	Moderate
Anwar et al. (2024) [16]	3	1	1	5/9	Moderate
Anwar et al. (2024) [17]	2	0	1	3/9	Low
Anwar et al. (2025) [18]	2	0	1	3/9	Low
Bhatt et al. (2019) [19]	2	0	1	3/9	Low
Bovonratwet (2022) [20]	4	1	1	6/9	Moderate
Bovonratwet (2024) [21]	3	1	1	5/9	Moderate
Cha et al. (2021) [23]	2	0	1	3/9	Low
Cha et al. (2021) [24]	2	0	1	3/9	Low
Cha et al. (2021) [58]	2	0	1	3/9	Low
Cha et al. (2022) [22]	4	2	1	7/9	High
Chen et al. (2021) [25]	1	0	1	2/9	Low
Chen et al. (2022) [26]	3	0	1	4/9	Moderate
Jacob et al. (2023) [28]	4	0	1	5/9	Moderate
Jacob et al. (2024) [27]	4	2	1	7/9	High
Karhade et al. (2021) [29]	4	1	2	7/9	High
Karhade et al. (2021) [30]	4	2	2	8/9	High
Kasir et al. (2025) [31]	3	2	2	7/9	High
Khechen et al. (2018) [32]	3	0	1	4/9	Moderate
Korsun et al. (2024) [33]	4	0	1	5/9	Moderate
Lightsey et al. (2022) [34]	3	0	1	4/9	Moderate
Lynch et al. (2021) [35]	3	0	1	4/9	Moderate
Lynch et al. (2022) [36]	4	0	0	4/9	Moderate
Mazur-Hart et al. (2025) [37]	4	1	1	6/9	Moderate
Merrill et al. (2018) [38]	4	0	1	5/9	Moderate
Mohanty et al. (2023) [39]	4	2	2	8/9	High
Nie et al. (2023) [40]	4	2	1	7/9	High
Nie et al. (2023) [41]	4	0	1	5/9	Moderate
Nie et al. (2023) [42]	4	0	1	5/9	Moderate
Nie et al. (2023) [43]	4	0	1	5/9	Moderate
Nolte et al. (2021) [44]	4	1	2	7/9	High
Nolte et al. (2021) [59]	4	1	1	6/9	Moderate
Oris et al. (2026) [45]	3	1	2	6/9	Moderate
Parrish et al. (2022) [46]	3	1	2	6/9	Moderate
Roca et al. (2024) [47]	3	1	2	6/9	Moderate
Rubery et al. (2019) [48]	4	2	1	7/9	High
Shahi et al. (2022) [49]	4	1	2	7/9	High
Song et al. (2024) [50]	3	0	2	5/9	Moderate
Turcotte et al. (2023) [51]	3	1	2	6/9	Moderate
Varlotta et al. (2020) [52]	3	0	1	4/9	Moderate
Virk et al. (2020) [53]	4	0	1	5/9	Moderate
Virk et al. (2020) [54]	4	0	1	5/9	Moderate
Ward et al. (2025) [55]	4	0	2	6/9	Moderate
Wolf et al. (2024) [56]	4	1	2	7/9	High
Zhang et al. (2023) [57]	4	2	2	8/9	High

Clinical Interpretability of PROMIS

Clinical interpretability through MCID analysis was reported in approximately 70% of included studies (32/45). However, the derivation of MCID thresholds was not consistently reported, and thresholds were not uniformly defined across cohorts, which may limit the comparability of clinically meaningful improvement. MCID was most frequently applied to PROMIS PF, followed by pain-related domains such as pain interference and pain intensity. In many studies, MCID analysis was used to report the proportion of patients achieving clinically meaningful improvement rather than relying solely on mean score changes, reflecting an emphasis on clinically relevant outcomes over statistical significance.

Formal validity assessment was less common. Only a minority of studies (8/45) used PROMIS specifically for validity testing, most commonly through construct or concurrent validity analyses comparing PROMIS domains with established legacy outcome measures. Approximately one-quarter of studies (11/45) reported correlation analyses with legacy measures, most frequently comparing PROMIS domains with instruments such as the ODI, VAS, and SF-12 or Veterans RAND 12-Item Health Survey (VR-12) physical component scores. When assessed, PROMIS PF and pain domains generally demonstrated moderate to very strong correlations with these established measures across both baseline and postoperative time points. In a subset of studies, PROMIS completion time was shorter than that of legacy measures and was not associated with PROMIS score values.

Predictive Utility and Modifiers of Improvement

Beyond outcome measurement, PROMIS was also used to evaluate predictors of postoperative recovery. While the majority of studies (29/45) employed PROMIS domains as primary outcome measures, a smaller subset (16/45) utilized PROMIS as a predictive instrument to examine whether baseline scores forecasted postoperative improvement, likelihood of achieving MCID, or downstream outcomes such as revision, opioid utilization, or recovery trajectory.

Among studies evaluating predictive performance, baseline symptom severity was among the most frequently reported predictors of postoperative PROMIS outcomes and clinically meaningful improvement. Worse baseline PROMIS PF, higher PROMIS PI, and greater preoperative pain burden were commonly associated with larger postoperative improvements or achievement of MCID. Additional predictors included demographic and health-related characteristics such as body mass index, comorbidity burden, and duration of symptoms, as well as psychosocial variables, including baseline depression severity and socioeconomic indicators such as neighborhood deprivation or insurance status. Structural and disease-specific characteristics, including disc herniation size, canal compromise, and muscle quality measures, were also reported to influence recovery in selected cohorts. In several studies, early postoperative PROMIS scores were shown to predict longer-term functional outcomes and recovery trajectories, and some cohorts demonstrated that intermediate PROMIS-PF change could differentiate patients who later required revision surgery.

Approximately half of the studies (23/45) identified patient-level or clinical factors that modified PROMIS outcomes or the likelihood of achieving clinically meaningful improvement. The most commonly reported modifiers related to baseline symptom burden include baseline PROMIS scores, baseline pain severity, depression severity, and sleep disturbance. Several studies demonstrated that patients with worse preoperative status experienced greater absolute postoperative improvement. Additional modifiers included demographic and health characteristics such as age, body mass index, and comorbidity burden, as well as clinical variables including symptom duration, spinal pathology, spinopelvic alignment, and surgical approach. Contextual and behavioral influences were also described in several cohorts, including opioid exposure and socioeconomic indicators such as neighborhood deprivation and insurance status. Muscle health parameters associated with sarcopenia were also reported as modifiers in some studies. Importantly, these factors appeared to influence the magnitude or timing of postoperative improvement more often than whether improvement occurred at all, suggesting that PROMIS outcomes capture meaningful variability in recovery trajectories following lumbar decompression.

Discussion

This systematic review synthesized the current literature evaluating the utilization of PROMIS instruments in patients undergoing lumbar decompression surgery, revealing several notable patterns. PROMIS PF was the most consistently reported domain across the included studies, reflecting the significant and central role that PF plays in functional recovery following decompressive spinal procedures [11]. Across the included studies, the PROMIS domains generally demonstrated postoperative improvement following lumbar decompression, with improvements being seen in short-term, mid-term, and long-term follow-up periods. A smaller subset of studies also used PROMIS as predictive tools to examine recovery trajectories and clinically meaningful improvement. However, the studies demonstrated substantial heterogeneity in terms of PROMIS domain selection, outcome reporting approaches, and analytical methodology, highlighting a continued limitation in direct comparability across cohorts.

Interpretation of Key Findings

The predominance of PROMIS PF in the lumbar decompression literature likely reflects the longstanding emphasis on restoring mobility and daily functioning following spine surgery. Traditionally, surgical outcomes in patients with lumbar spine conditions have been assessed using legacy instruments such as the ODI, VAS, and Short Form Health Surveys [5,6]. Due to the known limitations of these legacy instruments [7], PROMIS instruments have gained traction due to their improved measurement efficiency and standardized scoring framework [8]. Previous studies have spotlighted the strong psychometric performance of PROMIS instruments in patients with spinal disorders and have also shown that PROMIS PF correlates closely with traditional disability measures such as the ODI [11,13]. Notably, computer-adaptive testing has enabled PROMIS instruments to achieve comparable or even superior measurement precision while using fewer questionnaire items, thereby reducing respondent burden in both clinical and research settings [10]. Thus, the predominance of PROMIS PF in the lumbar decompression literature examined in this review likely reflects both the importance of functional recovery following surgery and the increasing adoption of PROMIS instruments in modern spine outcomes assessment.

The consistent postoperative improvement seen across PROMIS domains in this review highlights the responsiveness of these instruments to clinical recovery following lumbar decompression surgery. Previous research has shown that PROMIS measures are capable of detecting longitudinal change in patients with spinal disorders and strongly correlate with the well-known established outcome instruments, like ODI and NDI [11,13]. This review’s findings expand on this existing evidence by demonstrating that PROMIS responsiveness has been well-documented across a variety of decompression procedures and degenerative lumbar conditions. In fact, postoperative improvements in PROMIS domains were frequently seen early after surgery, with multiple studies demonstrating gains by six weeks postoperatively. These early improvements in functional and pain-related PROMIS scores are consistent with the underlying mechanism behind decompression procedures, which serve to relieve the neural compression that is responsible for symptoms such as radiculopathy and neurogenic claudication [3,4]. Altogether, these observations suggest that PROMIS instruments may be particularly useful for capturing both early postoperative recovery and longer-term functional outcomes following lumbar decompression.

Comparison With Previous Literature

These findings can also be understood in the context of prior systematic reviews examining PROMIS in broader spine surgery populations [60,61]. Haws et al. reported that early PROMIS literature in spine populations was largely centered on validation and correlations with legacy outcome measures, with only a minority of studies using PROMIS as an outcome measure itself [60]. Likewise, Ziedas et al. demonstrated that PROMIS PF compares favorably with legacy PROMs in spine surgery, particularly with respect to correlation strength, efficiency, and reduced survey burden [61]. In comparison, the present review provides a more procedure-specific synthesis focused on lumbar decompression and shows that PROMIS is not only being used for validation, but is increasingly being applied to assess postoperative responsiveness, clinically meaningful improvement, and prognostic utility [60,61]. Together, these findings suggest that the role of PROMIS in spine surgery has evolved from initial psychometric validation toward broader use in longitudinal outcome assessment and clinical decision-making [60,61]. Importantly, while prior reviews primarily emphasized the validity and efficiency of PROMIS instruments, the present review highlights their expanding role in assessing postoperative responsiveness and prognostic stratification within lumbar decompression populations, despite ongoing heterogeneity in study design and outcome reporting [60,61].

Prognostic Utility of PROMIS

In addition to measuring postoperative change, current literature suggests that PROMIS may also offer meaningful prognostic information regarding lumbar spine surgery [12]. Multiple studies in this review evaluated PROMIS scores as predictors of postoperative outcomes, with baseline PROMIS PF and PROMIS PI scores frequently being associated with the likelihood of achieving clinically meaningful improvements. These findings are in line with prior work, which has indicated that preoperative PROMIS scores may help predict the postoperative recovery in patients undergoing lumbar spine procedures [12]. Particularly, poorer baseline PROMIS PF scores have been associated with a higher probability of achieving MCID after surgery [12]. This finding may be reflective of the greater potential for improvement in patients with more severe preoperative functional limitations. Overall, these findings suggest that PROMIS can not only serve as an outcome assessment tool but may also be useful for risk stratification and preoperative counseling. In clinical settings, the ability to identify patients who are more likely to experience substantial postoperative improvement could support more informed shared decision-making and help establish realistic expectations for recovery following lumbar decompression [12,59].

Clinical Implications

The integration of PROMIS into routine clinical practice may also have important implications for spine surgery outcome assessment. The use of computer-adaptive testing allows for efficient and low-burden data collection, making PROMIS particularly well-suited for incorporation into busy clinical workflows. In addition to facilitating standardized longitudinal outcome tracking, PROMIS measures may support real-time clinical decision-making by providing insights into patient recovery trajectories and risk profiles, consistent with prior work using large clinical registries to predict patient-specific outcomes in spine surgery [62]. This may enhance preoperative counseling, enable more individualized postoperative management, and improve communication between patients and providers by offering a more comprehensive, patient-centered assessment of recovery beyond traditional metrics.

Methodological Heterogeneity and Outcome Variability

Another key finding from this review is the considerable methodological heterogeneity existing across the current literature examining PROMIS outcomes following lumbar decompression. Studies varied substantially in terms of the PROMIS domains, follow-up periods, and analytical approaches used to assess postoperative improvement. While PROMIS PF was reported in the vast majority of included studies, mental health-related domains such as depression, anxiety, and sleep disturbance were less frequently assessed. This difference likely reflects the traditional focus of spine surgery research on physical disability and pain outcomes. However, the PROMIS framework was designed specifically to incorporate multiple dimensions of health, including physical, mental, and social well-being [8]. Emerging evidence indicates that psychosocial factors, including depression, anxiety, and socioeconomic status, can influence postoperative outcomes in spine surgery populations [57,63,64]. Thus, expanding the use of these broader PROMIS domains in future research may allow for a more comprehensive understanding of patient recovery and the factors contributing to variability in postoperative outcomes.

Beyond the heterogeneity present in PROMIS domain selection, differences in how clinically meaningful improvement was defined also represented a limitation within the current lumbar decompression literature. While many studies reported MCID analyses, the thresholds used were not standardized and were instead derived using varying methodological approaches across cohorts. These differences reduce the comparability between studies and thus complicate the interpretation of whether reported postoperative changes represent similar levels of patient-perceived benefit. Previous research has emphasized that changes in PROMIS scores should be interpreted relative to clinically meaningful thresholds rather than just relying solely on statistical significance [9,13,65]. Future studies would benefit from more transparent and standardized reporting of MCID derivation methods, which would improve interpretability and facilitate more meaningful comparisons across cohorts.

PROMIS outcomes also appeared to vary depending on a range of patient-level and clinical characteristics across the included studies. Variables such as age, body mass index, comorbidity burden, baseline depression severity, symptom duration, and preoperative pain burden were associated with differences in postoperative PROMIS trajectories and/or the likelihood of achieving clinically meaningful improvement. These observations suggest that recovery after lumbar decompression is not just determined by operative pathology, but also by the complex interaction of biological, psychosocial, and socioeconomic factors. Previous literature has demonstrated how psychological distress and socioeconomic disadvantage are associated with differences in postoperative recovery and patient-reported outcomes [57,64,66-68]. In this context, PROMIS may be particularly valuable because its multidomain structure enables outcome assessment to extend beyond just physical function and more comprehensively include the broader determinants of recovery after lumbar spine surgery. In parallel, emerging research has explored the application of machine learning approaches to predict postoperative patient-reported outcomes in spine surgery, highlighting a growing interest in personalized and data-driven models of outcome prediction [69].

Strengths

This review has several strengths. To our knowledge, it represents one of the first systematic reviews specifically examining the use of PROMIS outcome measures in lumbar decompression surgery. In contrast to previous studies that have assessed PROMIS performance across broader spine surgery populations, this review focused exclusively on decompression procedures for degenerative lumbar disease, enabling a more procedure-specific analysis. Furthermore, the review evaluated multiple aspects of PROMIS utilization, including postoperative responsiveness, achievement of clinically meaningful improvement, prognostic applications, and correlations with established legacy outcome measures. By examining these different dimensions, this review provides a more comprehensive overview of how PROMIS instruments are currently being applied within the lumbar decompression literature.

Limitations

Several limitations should also be considered when interpreting these findings. Firstly, most of the included studies were retrospective cohort studies conducted at single institutions, which introduces potential selection bias, limits generalizability, restricts the ability to establish causal relationships, and increases susceptibility to unmeasured confounding that may influence observed PROMIS outcomes. Additionally, the predominance of retrospective designs may contribute to variability in data completeness, follow-up consistency, and outcome reporting, further limiting the robustness and comparability of findings across studies. Secondly, most studies originated from the United States, resulting in very limited representation from other types of healthcare systems. Thirdly, substantial heterogeneity in PROMIS domains assessed, follow-up intervals, and analytical methodologies limited comparability across studies and precluded quantitative pooling of results, necessitating a qualitative synthesis. Fourthly, many studies focused mainly on physical function and pain domains, while psychological and social health constructs were evaluated less consistently. Additionally, the overall moderate methodological quality and predominance of retrospective cohort designs, as identified through risk-of-bias assessment, further limit the strength of causal inference and generalizability of the findings. Finally, variability in MCID thresholds and outcome reporting approaches limits direct comparison of clinically meaningful improvement across cohorts.

Despite these limitations, the findings of this review suggest that PROMIS has become an increasingly important tool for evaluating outcomes following lumbar decompression surgery. PROMIS PF and pain-related domains appear especially well-established and consistently demonstrate responsiveness to postoperative improvement. Moreover, emerging evidence indicates that PROMIS scores may offer useful prognostic information regarding postoperative recovery trajectories. Future research would benefit from greater standardization in PROMIS domain selection, MCID reporting, and analytical methodologies, as well as an increased incorporation of prospective and multicenter study designs. These efforts may further clarify the role of PROMIS as a standardized and efficient approach for outcome assessment in lumbar spine surgery.

## Conclusions

PROMIS outcome measures have become an increasingly important tool for assessing recovery in patients following lumbar decompression surgery. Across the existing literature, PROMIS PF and pain-related domains were the most frequently used and consistently demonstrated responsiveness to postoperative improvement across short-, mid-, and long-term follow-up periods. PROMIS measures also showed meaningful correlations with legacy outcome instruments and, in a subset of studies, demonstrated potential utility as predictors of postoperative recovery and clinically meaningful improvement. However, the current literature remains methodologically heterogeneous, particularly in terms of PROMIS domain selection, MCID reporting, and analytical approaches, and is largely composed of observational study designs. Overall, the findings of this review suggest that PROMIS represents a promising and increasingly utilized approach for outcome assessment in lumbar decompression surgery, while highlighting the need for greater methodological standardization and broader incorporation of multidimensional PROMIS domains in future research.

## Appendices

Appendix A: Full PubMed search strategy

The following search strategy was used in PubMed:

(("PROMIS"[All Fields] OR "Patient-Reported Outcomes Measurement Information System"[All Fields]) AND ("lumbar"[All Fields] OR "lumbar spine"[All Fields] OR "lumbar disc"[All Fields] OR "spinal stenosis"[All Fields]) AND ("decompression"[All Fields] OR "laminectomy"[All Fields] OR "discectomy"[All Fields] OR "microdiscectomy"[All Fields])) AND (Humans[Filter]) AND (English[Filter]) AND ("2010/01/01"[Date - Publication] : "2026/01/31"[Date - Publication]) AND (adult[MeSH Terms])

Filters applied: Humans, Adults (≥18 years), English language, and publication dates from 2010 to 2026.

Appendix B. Full Embase search strategy

The following search strategy was used in Embase:

((PROMIS OR "Patient-Reported Outcomes Measurement Information System") AND (lumbar OR "lumbar spine" OR "lumbar disc" OR "spinal stenosis") AND (decompression OR laminectomy OR discectomy OR microdiscectomy)).mp.

Limits applied: Humans, English language, and publication dates from 2010 to 2026.

Appendix C. Full Scopus search strategy

The following search strategy was used in Scopus:

TITLE-ABS-KEY (PROMIS OR "Patient-Reported Outcomes Measurement Information System") AND TITLE-ABS-KEY (lumbar OR "lumbar spine" OR "lumbar disc" OR "spinal stenosis") AND TITLE-ABS-KEY (decompression OR laminectomy OR discectomy OR microdiscectomy)

Limits applied: Humans, English language, and publication dates from 2010 to 2026.
